# Characterization of a MexAB-OprM efflux system necessary for productive metabolism of *Pseudomonas azelaica* HBP1 on 2-hydroxybiphenyl

**DOI:** 10.3389/fmicb.2013.00203

**Published:** 2013-07-19

**Authors:** K. Czechowska, C. Reimmann, J. R. van der Meer

**Affiliations:** Department of Fundamental Microbiology, University of Lausanne, Bâtiment Biophore, Quartier UNIL-SorgeLausanne, Switzerland

**Keywords:** pollutant toxicity, biodegradation, aromatic compound, coal desulfurization

## Abstract

*Pseudomonas azelaica* HBP1 is one of the few bacteria known to completely mineralize the biocide and toxic compound 2-hydroxybiphenyl (2-HBP), but the mechanisms of its tolerance to the toxicity are unknown. By transposon mutant analysis and screening for absence of growth on water saturating concentrations of 2-HBP (2.7 mM) we preferentially found insertions in three genes with high homology to the *mexA, mexB*, and *oprM* efflux system. Mutants could grow at 2-HBP concentrations below 100 μM but at lower growth rates than the wild-type. Exposure of the wild-type to increasing 2-HBP concentrations resulted in acute cell growth arrest and loss of membrane potential, to which the cells adapt after a few hours. By using ethidium bromide (EB) as proxy we could show that the mutants are unable to expel EB effectively. Inclusion of a 2-HBP reporter plasmid revealed that the wild-type combines efflux with metabolism at all 2-HBP concentrations, whereas the mutants cannot remove the compound and arrest metabolism at concentrations above 24 μM. The analysis thus showed the importance of the MexAB-OprM system for productive metabolism of 2-HBP.

## Introduction

Bacteria degrading xenobiotic compounds often face the difficulty that the chemicals are not only very difficult to metabolize but are also very toxic (van der Meer, 2006). Toxicity often occurs as a direct consequence of the xenobiotic compound's hydrophobicity causing it to partition into the cellular membranes (Sikkema et al., 1995; Denich et al., 2003). Mechanistic studies of chemical toxicity to biological membranes have underscored two general processes: baseline toxicity or narcosis, and uncoupling (van Wezel and Opperhuizen, 1995). Narcosis refers to the interference of membrane structure and function through partitioning of a chemical in its neutral state into the membrane (Vighi et al., 2009). Documented effects include membrane expansion or swelling (Sikkema et al., 1995; Neumann et al., 2005), increased membrane rigidity (Heipieper et al., 2003), as well as to lower permeability for ions (van Wezel and Opperhuizen, 1995). Additional more specific and selective effects may arise when organic compounds disrupt the proton or ion gradient across energy-transducing membranes, a process known as uncoupling (Escher et al., 2001). Partitioning of hydrophobic organic compounds into cellular membranes can be described by the octanol-water coefficient (Escher et al., 2008) as well as by their dissociation constants (Escher et al., 2000, 2002). Such calculations predict accumulations for strongly lipophilic compounds of thousandfold or more in the membrane compared to the cytoplasm or extracellular environment (Sikkema et al., 1995).

Bacteria can withstand a certain level of membrane insults through tolerance or resistance mechanisms. These include restructuring of the cell membrane and *cis-trans* isomerization reactions at the level of the phospholipid fatty acid chains (Heipieper et al., 2003), or repulsion and active efflux of solvent molecules from the cell (Ramos et al., 2002). Bacterial defense against toxic assaults is assumed to be evolutionary very ancient, and at least five different families of multi-drug resistance (MDR) pumps are understood (Alvarez-Ortega et al., 2013). These include ATP-Binding Cassette (ABC) superfamily transporters, that rely on ATP hydrolysis; the Major Facilitator Superfamily (MFS), the Small Multidrug Resistance (SMR) and the Resistance/Nodulation/Division (RND) superfamilies, which are dependent on proton motive force; and finally, the Multidrug And Toxic compound Extrusion (MATE) superfamily, operating mostly in grampositive bacteria and dependent on proton/sodium antiport activity (Alvarez-Ortega et al., 2013). Mostly, tolerance mechanisms against toxicity of metabolizable xenobiotic compounds have been deduced from a few model systems, such as the SprABC efflux system of *Pseudomonas putida* S12 to styrene (Kieboom et al., 1998), and the TtgABC/TtgDEF and TtgGHI systems of *P. putida* DOT-T1E to toluene and other solvents (Rojas et al., 2001; Ramos et al., 2002). All these systems belong to the RND-superfamily, which also includes the well-characterized MexAB-OprM antimicrobial resistance efflux system of *P. aeruginosa* (Li et al., 1998).

The major goal of the underlying work was to identify the basis of resistance to toxicity of 2-hydroxybiphenyl (2-HBP) in *Pseudomonas azelaica* HBP1 (Kohler et al., 1988). 2-HBP is a volume chemical with bactericidal and biocidal properties similar to triclosan (Schweizer, 2001), that is applied in industry, personal health care products and household disinfectants, as well as pesticides and fungicides (Jiang et al., 2010). As a consequence of its widespread use, 2-HBP can be found in persistent low quantities in sewage effluents (Yu et al., 2011). In addition, 2-HBP is the most important byproduct of biodesulfurization of oil and coals, formed through microbial conversion of dibenzothiophene (Gunam et al., 2013), causing both acute and chronic toxicity to the microbial strains in the conversion process (Alves and Paixao, 2011). As substituted phenol 2-HBP is a weak hydrophobic acid that can occur both in its protonated and dissociated form; the dissociated form being able to take up protons from the cytoplasmic interior across the membrane to the extracellular environment. 2-HBP may therefore be expected to have both baseline toxicity and uncoupling effects, as well as causing direct denaturation to proteins. Bacterial degradation of 2-HBP is relatively rare and isolates recovered from the environment that metabolize 2-HBP cannot withstand high 2-HBP concentrations (Czechowska, unpublished). One of the few bacteria known to completely metabolize 2-HBP is *P. azelaica* HBP1 (recently taxonomically renamed as *Pseudomonas nitroreducens*). Strain HBP1 was isolated from a wastewater treatment plant and can efficiently grow on 2-HBP up to the maximum aqueous soluble concentration of 2.7 mM (Kohler et al., 1988). The strain metabolizes 2-HBP via the action of three specific enzymes named HbpA (a hydroxylase), HbpC (an extradiol dioxygenase), and HbpD (a hydrolase), that lead to the formation of benzoate and 2-hydroxy-2,4-pentadienoic acid, which then subsequently are taken up in the regular metabolic pathways (Kohler et al., 1988, 1993; Jaspers et al., 2001). The metabolic pathway is under control of the HbpR protein, which activates transcription of the *hbpCA* and *hbpD-genes* in the presence of 2-HBP from two promoters named P_C_ and P_D_, respectively (Jaspers et al., 2001). One of the important questions in 2-HBP metabolism thus concerns the mechanism(s) that make(s) strain HBP1 so resistant to 2-HBP. In order to study this question we used transposon mutagenesis of strain HBP1 to identify mutants that are unable to grow in the presence of 2-HBP. The exact insertion positions of the transposons were determined and mapped on a draft genome sequence of *P. azelaica*. Toxicity effects of 2-HBP for wild-type and mutant *P. azelaica* were analyzed by flow cytometry (FC), in presence or absence of specific physiological dyes to understand the possible mechanism of action. Uptake of 2-HBP in wild-type and mutant strains was followed indirectly, by using strains equipped with an intracellular bioreporter system that produces GFP upon contact to 2-HBP.

## Materials and methods

### Strains and culture conditions

*P. azelaica* HBP1 was used as wild-type strain and is able to completely mineralize 2-HBP (Kohler et al., 1988). *P. azelaica* strains were cultured at 30°C in liquid Pseudomonas Minimal Medium (PMM) (Gerhardt et al., 1981), or M9 minimal medium (Sambrook and Russell, 2001) amended with 5 mM sodium succinate and/or 2-HBP in different concentrations (Sigma-Aldrich, Switzerland). Tn5 mutants of strain HBP1 were cultured in the presence of 50 μg/mL of kanamycin (Km). To maintain the 2-HBP reporter plasmid pME6012_hbpR_gfp (see below) 10 μg/mL tetracycline (Tc) was added to the culture medium. As solid medium for *P. azelaica* strains we used Nutrient Agar (NA) or PMM agar (15 g/L, DIFCO BactoAgar; Brunschwig DB Difco, Basel, Switzerland) with either 5 mM sodium succinate or 2.7 mM 2-HBP, and with or without Km or Tc as indicated above. *Escherichia coli* DH5α (Sambrook and Russell, 2001) and S17-1/λpir (Simon et al., 1993) were cultured at 37°C in liquid Luria Bertani (LB) Broth or on LB agar; if required, under inclusion of the appropriate antibiotics.

### Selection of mutants unable to grow with 2-HBP

Tn*5* insertion mutants were generated by biparental matings between *E. coli* BW20767/pRL27 (Larsen et al., 2002) and *P. azelaica* HBP1 with selection on NA containing 50 μg/ml Km and 10 μg/ml chloramphenicol, to counterselect against the *E. coli* donor. About 10,000 mutant colonies, produced in eight independent mutagenesis experiments, were picked individually and tested by replica plating for growth on M9 minimal medium with 5 mM succinate vs. 2.7 mM 2-HBP as sole carbon source. Mutants which failed to develop colonies on 2-HBP after 3 days but were growing on succinate, were investigated further. To determine the transposon insertion site in these mutants, chromosomal DNA was extracted as described (Gamper et al., 1992), subjected to restriction with SacII, self-ligated and introduced into *E. coli* S17-1/λpir by electroporation, with selection for Km resistance. After isolation of the plasmid, the Tn*5* insertion sites were determined by Sanger sequencing using the transposon-specific primer tnpRL17-1 (5′-AACAAGCCAGGGATGTAACG-3′) (Larsen et al., 2002). Sequences were mapped onto a draft *P. azelaica* genome (van der Meer, unpublished) using BLASTN and further interpreted according to database homologies of the predicted gene functions into which the insertions had occurred.

### Cell counting by flow cytometry

Total numbers of cells in culture were counted with a BD LSR Fortessa (BD Biosciences, Erembodegem, Belgium) equipped with 3-lasers (Blue = 488 nm, 50 mW; Red = 640 nm, 40 mW; UV = 355, 20 mW). Cells were hereto fixed with 4 g/L sodium azide (Sigma-Aldrich, Switzerland) for 1 h at 4°C and stained with 100-fold diluted SYBRGreen I solution (Invitrogen, Switzerland) for 15 min in the dark at room temperature. A fixed sample volume (200 μL) was aspirated by a High Throughput Screening device (BD Biosciences) at a flow rate of 1 μL/s. Culture samples exceeding 1000 events/s were further diluted in filtered PMM. Data were acquired in the FITC-channel and processed using the BD software DIVA (version 6.2, BD Biosciences). Side and forward scatter were set to 250 to reduce background noise.

Cell division rates were calculated from the increase of cell numbers in culture over time measured by FC, and converted to maximum specific growth rates (μ) by multiplying by ln2 (Czechowska et al., 2013).

### Membrane transport inhibition assays

Membrane transport was studied using ethidium bromide (EB) as proxy. HBP1 wild-type cells or mutants were grown until mid exponential phase on 5 mM succinate, then sampled and stained with SYTO9 (5 μM, Invitrogen) plus EB (10 μM) for 10 min in the dark, either in absence or in the presence of potential membrane uncouplers. SYTO9 and EB fluorescence in individual cells were then immediately measured using FC, as above. As membrane uncouplers we used carbonyl cyanide-3-chlorophenyl-hydrazone (CCCP, at 15 μM), sodium azide (4 g/L), tetraphenylphosphonium (TPP, at 4 μM), valinomycin (at 1 μM), and ethylenediaminetetraacetic acid (EDTA, at 10 mM). All other compounds were obtained from Sigma-Aldrich (Switzerland). For EB efflux assays cells were grown until mid exponential phase on 5 mM succinate, stained with 10 μM EB for 10 min in the dark, centrifuged for 1 min at 13,000 g and resuspended in PMM without EB. The intensity of EB fluorescence was measured by FC after 0, 10, 20, 30, and 40 min.

### Bacterial membrane integrity and membrane potential

To assess 2-HBP toxicity we exposed *P. azelaica* HBP1 and mutant cells sampled from exponentially growing culture on PMM with succinate, for 1 or 3 h to 100 μM, 500 μM, or 1 mM 2-HBP. Effects were compared to cells taken from the same culture, but exposed for 1 and 3 h at 30°C to CCCP, sodium azide and valinomycin (concentrations above). Samples were then stained immediately with the BacLight Live/Dead Kit according to the protocol provided by the manufacturer (Invitrogen). Similarly, the cellular membrane potential was measured by staining with 50 nM final concentration 3,3′-diethyloxacarbocyanine iodide (DiOC_2_(3)), according to technical instructions of the supplier (Invitrogen). Fluorescence intensities were measured on a FACS Calibur flow cytometer (BD Biosciences) using FL1 (525 ± 15 nm) and FL3 (>650 nm) channels.

### Intracellular detection of 2-HBP using a pME6012_hbpR_gfp reporter plasmid

In order to test influx of 2-HBP into *P. azelaica* HBP1 and mutants we used a reporter plasmid that was previously developed for *E. coli* (Beggah et al., 2008). GFP production is here brought under control of the HbpR-and 2-HBP-dependent P_C_-promoter (Jaspers et al., 2001). The vector of this reporter plasmid (pHBP269A0) was changed to that of pME6012 (Heeb et al., 2000), to make it better compatible with *P. azelaica*. Hereto, the *hbpR-P_C_::gfp* gene fragment was recovered from pHBP269A0 by cutting with NheI and SalI, and ligated with pME6012 cut with NheI and XhoI. After ligation the plasmid was introduced into *E. coli* DH5α by heat shock transformation. Positive clones containing the new plasmid pME6012_hbpR_gfp were selected on LB agar containing 10 μg/mL Tc. The plasmid was isolated and verified by restriction enzyme digestion. Induction of GFP in *E. coli* (pME6012_hbpR_gfp) was verified by exposing the cells to 2.5 mM 2-HBP for 2 h (not shown). Purified pME6012_hbpR_gfp was then introduced by electroporation into *P. azelaica* HBP1 and the three selected Km-insertion mutants (*mexA::*Km, *mexB::*Km, *oprM::*Km).

### 2-HBP uptake experiments

Uptake of 2-HBP by strains HBP1, *mexA::*Km, *mexB::*Km, or *oprM::*Km was followed by measuring the GFP signal generated from the reporter plasmid pME6012_hbpR_gfp in the presence or absence of membrane uncouplers. Cells were hereto grown on PMM with 5 mM succinate until mid-exponential phase. Culture samples of 250 μL were transferred into wells of a 96-well black microtiter plate (Greiner Bio-One GmbH, Germany). Cells were incubated at 30°C with varying concentrations of 2-HBP (0, 2.4, 24, and 240 μM), upon which GFP fluorescence of the cultures was followed for 7 h. GFP fluorescence (at 520 nm) and culture turbidity (at 600 nm) were measured at regular time intervals in a FLUOstar Omega fluorimeter (BMG LABTECH SARL, France). GFP fluorescence values were normalized to the culture turbidity.

## Results

### Transposon insertions in the *mexAB-oprM* gene cluster

**Figure 1:**
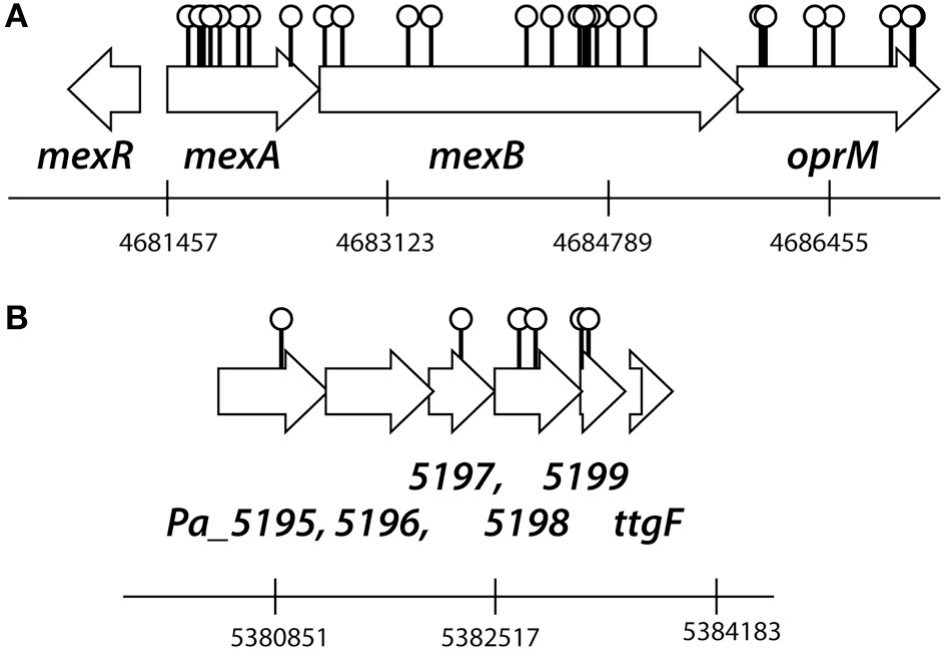
**Outline of the transposon insertion positions in the *P. azelaica* HBP1 genome resulting in abolished growth on 2-HBP. (A)** Insertions in the *mexAB-oprM* gene cluster. **(B)** Insertions in the putative ABC-type organic solvent resistance efflux system. Positioning and numbering of predicted *P. azelaica* genes according to a draft genome assembly (van der Meer, unpublished). See further Table S1 and Data File S1 for the DNA and amino acid sequences of the two regions of *P. azelaica*.

Replica plating of some 10,000 *P. azelaica* transposon mutants from eight independently created mutant libraries resulted in ~1% of colonies growing on succinate but unable to grow with 2.7 mM (460 mg/L) 2-HBP as a sole carbon source. The transposon insertion and flanking DNA sequences of 95 mutants unable to grow on 2-HBP were cloned and the insertion sites determined by sequence analysis and BLASTN comparison (Table S1, Data File S1). We found that 28 insertions had occurred at different positions in a gene cluster homologous to *mexAB-oprM* (9 insertions occurred in *mexA*, 12 in *mexB*, and 7 in *oprM*), specifying a multidrug resistance efflux pump (Figure 1). Percent amino acid similarities across the whole length between the *P. azelaica* and *P. aeruginosa* MexAB-OprM systems approached 87% (MexA), 96% (MexB), 89% (OprM), and 69% (MexR). Interestingly, no transposon insertions that caused failure to grow on 2-HBP were recovered in *mexR* (Figure 1), a presumed regulatory gene for *mexAB-oprM* expression. A further 6 had inserted into a cluster of six genes, encoding a putative ABC transport system providing resistance to organic compounds with over 90% amino acid similarity to Cluster of Orthologous Groups (COG) PA4452-4455 (Figure 1B). Insertions from the remaining mutants had occurred throughout the *P. azelaica* genome but in no further cases were found multiple times in the same gene. Intriguingly, no transposon mutants were recovered with insertions in the known *hbpR-CAD* genes for 2-HBP metabolism (Jaspers et al., 2001).

### 2-HBP toxicity for *P. azelaica* HBP1

**Figure 2:**
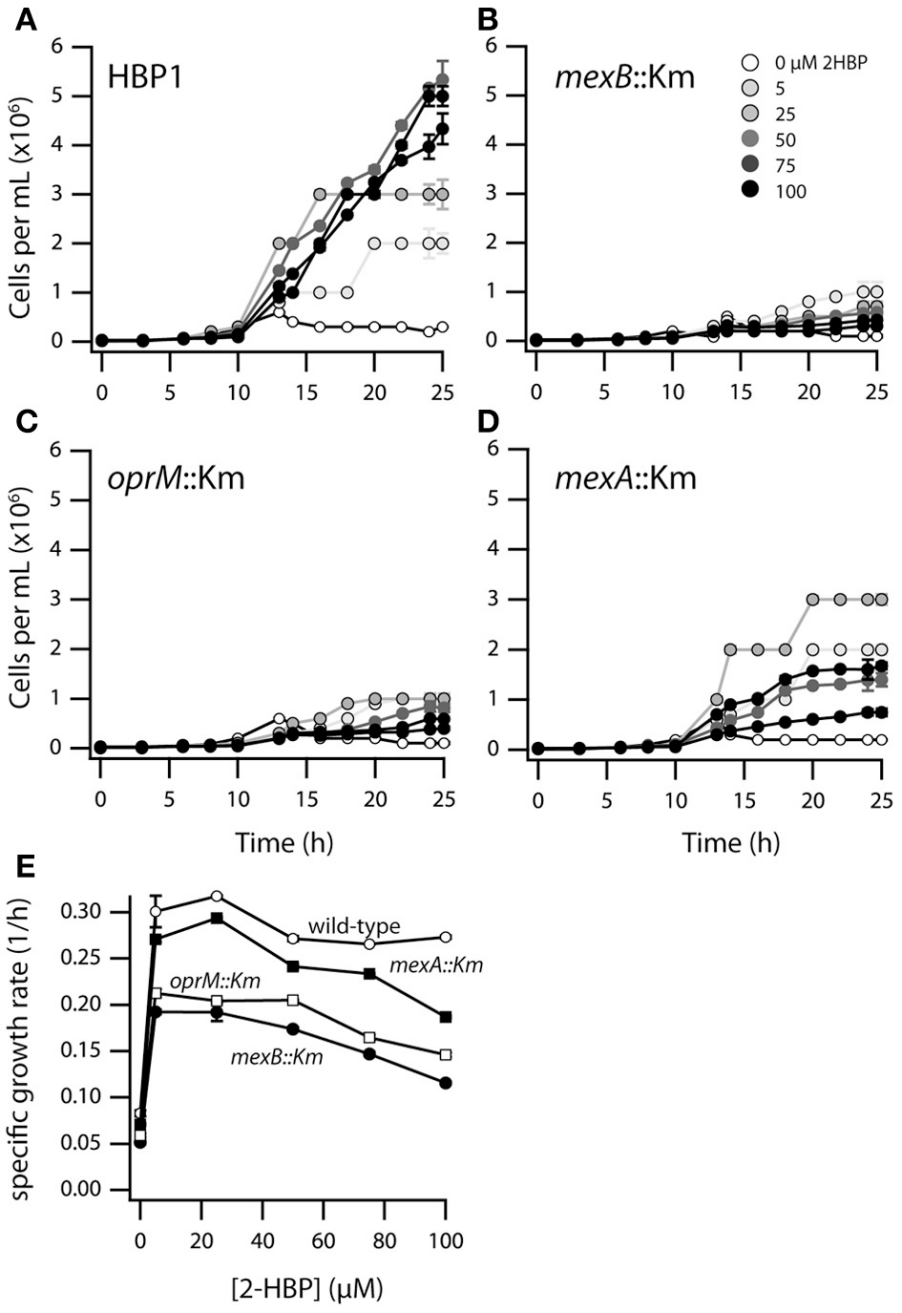
**Growth of *P. azelaica* HBP1 wild-type, *mexA::*Km, *mexB::*Km, and *oprM::*Km mutants on low concentrations of 2-HBP. (A–D)** Population growth over time as function of 2-HBP concentration. **(E)** Calculated maximum specific growth rates for wild-type or mutants as a function of 2-HBP concentration.

In order to better understand the effects of 2-HBP on the growth of wild-type HBP1 and three transposon mutants, one in each of the *mexAB* or *oprM* genes, we re-examined culture growth on 2-HBP in low concentrations between 0 and 100 μM (Figure 2). Population growth at these low concentrations was measured by FC counting of fixed and stained cells. Interestingly, although the three mutants did not grow on 2.7 mM 2-HBP, they did grow to some extent on 2-HBP concentrations up to 25 μM (Figures 2B–D), indicating they were unable to cope with toxicity exerted by high 2-HBP concentrations. Calculated maximum specific growth rates even at low 2-HBP concentrations were lower for all transposon mutants compared to HBP1 wild-type, with HBP1 *mexB*::Km and *oprM*::Km being the most severely affected, followed by HBP1 *mexA*::Km (Figure 2E).

**Figure 3:**
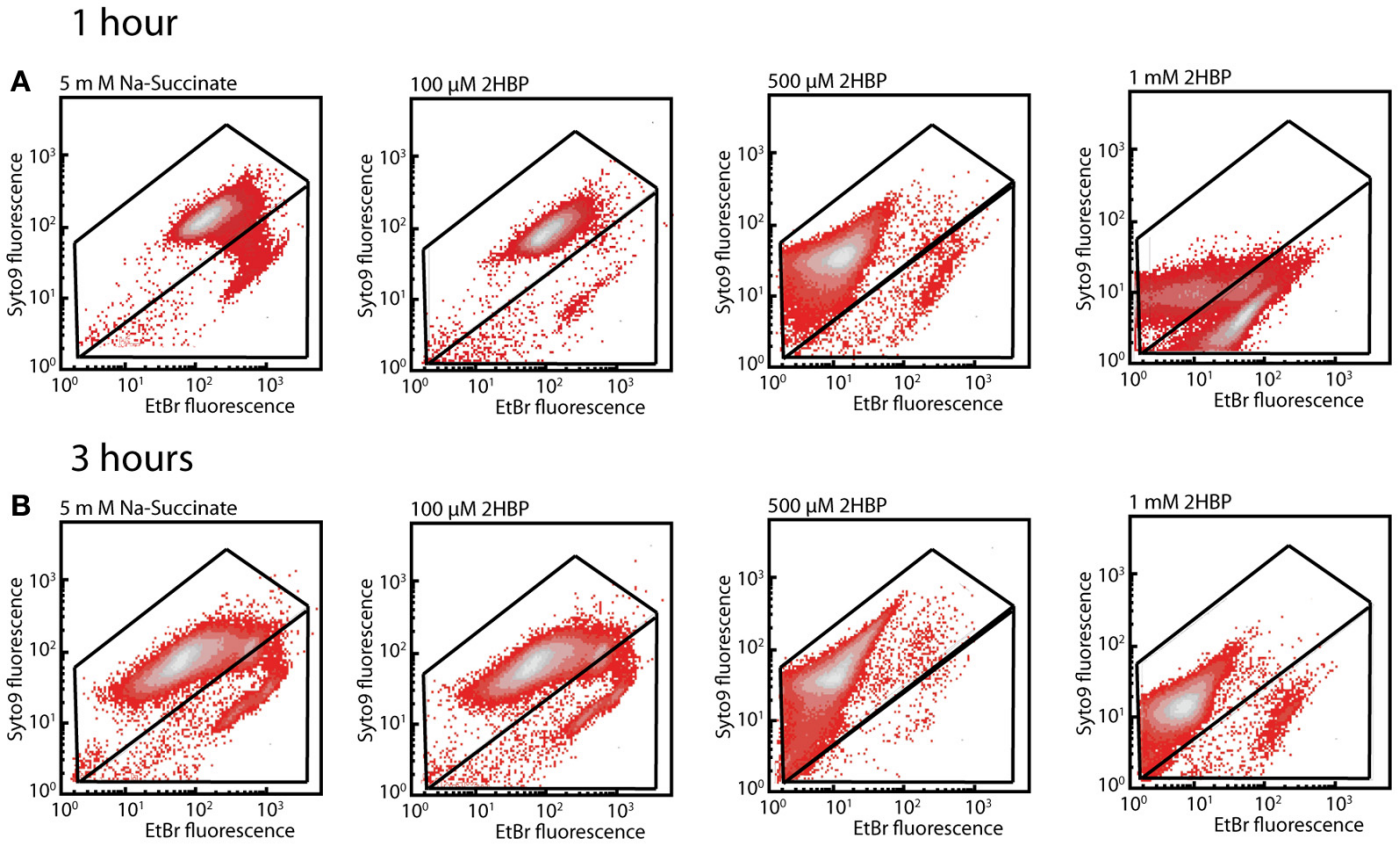
**Membrane damage in *P. azelaica* HBP1 wild-type cells as a function of exposure to 2-HBP. (A)** Exponentially growing cells on 5 mM sodium succinate exposed or not to 2-HBP at 100 μM, 0.5 and 1 mM concentrations. Cells sampled after 1 h exposure and stained with SYTO9 plus EB. **(B)** As for **(A)**, but sampled and stained after 3 h exposure.

In order to understand the possible mechanism of toxicity of 2-HBP to strain HBP1 we exposed cells from mid-exponential phase on sodium succinate to 100 μM, 500 μM, and 1 mM of 2-HBP for 1 and 3 h. Cells exposed to 100 or 500 μM stained after 1 and 3 h with SYTO9 plus EB or SYTO plus propidium iodide (PI; part of the Live/Dead stain) did not show any increase in EB fluorescence or in the proportion of injured/dead cells, compared to non-exposed cells (Figures 3, S1). In contrast, 1 h exposure to 1 mM 2-HBP resulted in a significant increase of the proportion of injured/dead cells, as well as a strong decrease in both EB, SYTO9, and PI fluorescence. This decrease is similar as what is observed in stationary phase cells and suggests a complete metabolic arrest of cells (not shown). Some recovery of EB, SYTO9, and PI signal in the live cell proportion occurred 3 h after exposure to 1 mM 2-HBP, suggesting that cells started to divide again (Figures 3, S1).

**Figure 4:**
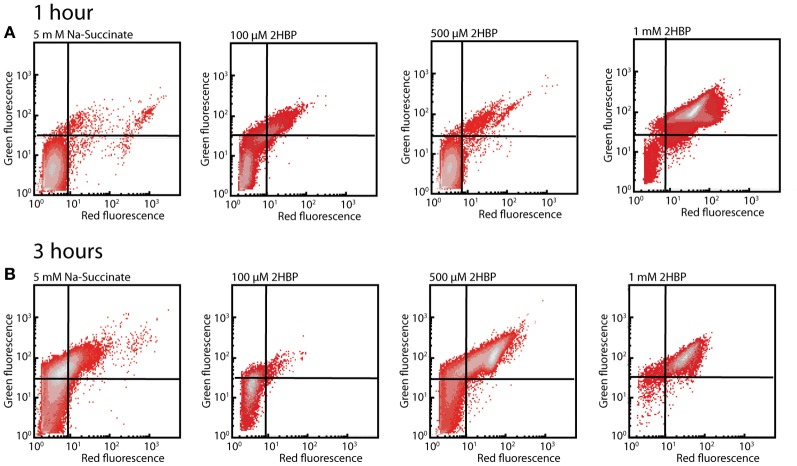
**Membrane potential loss in *P. azelaica* HBP1 wild-type cells after exposure to 2-HBP. (A)** Exponentially growing HBP1 cells on 5 mM sodium succinate exposed or not to 2-HBP at 100 μM, 0.5 or 1 mM for 1 h, after which they were sampled and stained with DiOC_2_(3). **(B)** As **(A)**, but after 3 h exposure. Populations displayed on density plots. Quadrants were set on the basis of cells non-exposed to 2-HBP or cells treated with sodium azide.

An increased red fluorescence intensity was observed of cells exposed to 500 μM and 1 mM 2-HBP stained with DiOC_2_(3) compared to the non-exposed cells, both after 1 h (Figure 4A) and 3 h (Figure 4B), suggesting that such cells have increased depolarization of their membranes as a consequence of being exposed to 2-HBP. The effect caused by 1 mM 2-HBP is comparable to the one observed when cells are exposed to 4 g per L of sodium azide (Figure S2). In both cases an increase of red fluorescence reflecting DiOC_2_(3) intracellular aggregation due to membrane potential loss is observed (Joux and Lebaron, 2000), suggesting that 2-HBP toxicity is exerted at the level of uncoupling. These data therefore indicated that also wild-type HBP1 experiences acute 2-HBP toxicity at higher concentrations but somehow can cope with this and resume growth. From the absence of growth on 2-HBP by the *mexAB-oprM* mutants we hypothesized that the MexAB-OprM system would be responsible for mitigating 2-HBP toxicity in the wild-type.

### EB monitored efflux of *P. azelaica* HBP1 Tn5 insertion mutants

**Figure 5:**
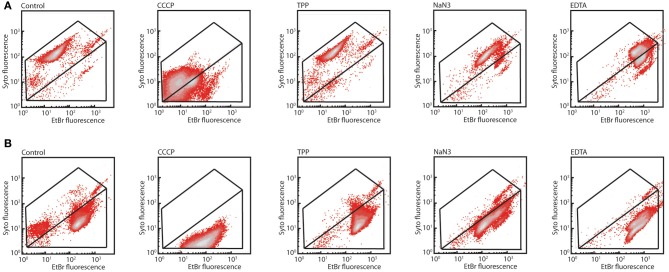
**Differential response of *P. azelaica* HBP1 wild-type or *mex/oprM* mutants to membrane energy uncouplers. (A)** Exponentially growing cells of HBP1 on 5 mM sodium succinate exposed or not to CCCP (15 μM), TPP (1 μM), sodium azide (4 g/L), or EDTA (10 mM), and stained with SYTO9 plus EB. **(B)** As **(A)**, but with *P. azelaica* HBP1 *mexA::*Km. Note that the *mexB::*Km and *oprM::*Km mutants are not shown but behaved very similarly.

In order to understand how the *mexAB-oprM* encoded system in *P. azelaica* would function to abolish 2-HBP toxicity, we used a previously developed EB equilibrium assay (Czechowska and van der Meer, 2012). This assay measures EB fluorescence in live cells exposed or not to membrane inhibitors under the premise that EB influx is spontaneous and efflux is energy dependent. Our hypothesis was that the MexAB-OprM system is an energy-dependent efflux system in strain HBP1 capable of expulsing EB. Interestingly, the staining patterns of the three mutants in all three treatments were similar but very distinct from the one of HBP1 wild-type (Figure 5 shows the results for wild-type and the *mexA::*Km mutant). In particular the SYTO9 fluorescence intensity was lower and independent of the treatment. EB fluorescence decreased in wild-type cells exposed to CCCP compared to the control, but increased after exposure to sodium azide and EDTA. Addition of TPP had no visible effect (Figure 5A). Under the assumption that EB influx would remain constant this suggested that efflux energy was interrupted by sodium azide, but not by TPP or CCCP. In contrast, EB fluorescence of the mutants was already 10 times higher, and SYTO9 fluorescence 10 times lower than wild-type in non-exposed and TPP-exposed cells. Addition of CCCP and sodium azide caused a slight decrease of EB fluorescence in mutant cells compared to the control. EDTA caused a further increase of EB fluorescence intensity in mutant cells (Figure 5B). Assuming that EB influx rates remain constant, the fact that higher equilibrium EB levels occurred in mutant cells and cellular inhibitors only exerted slight effects then suggests that EB efflux was impaired. This would be in agreement with the hypothesis that the *mexAB-oprM* system of *P. azelaica* HBP1 encodes a multi-drug efflux pump similar as in *P. aeruginosa* (Li et al., 1998).

**Figure 6:**
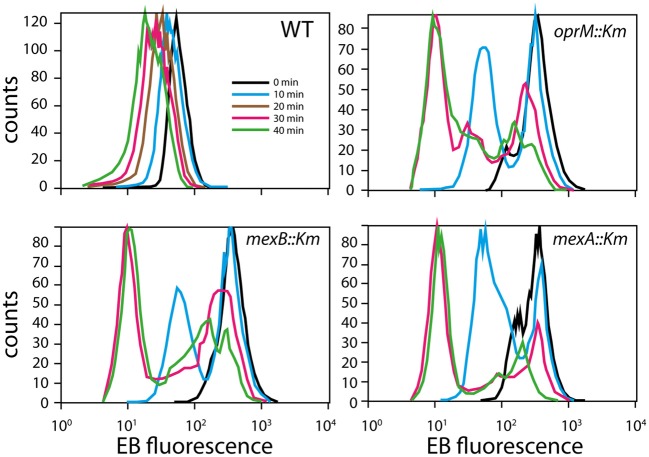
**EB efflux in *P. azelaica* HBP1 and the selected transposon mutants.** Cells were loaded with 10 μM EB and then resuspended in growth medium w/o EB. Graphs display the distribution of EB fluorescence levels in cells at times 0, 10, 20 (for wild-type only), 30, and 40 min.

To test impaired efflux directly, cells were preloaded with EB and examined for their ability to efflux the dye over time (Figure 6). EB fluorescence decreased in HBP1 wild-type cells over time with an apparent zeroth order constant of −1.15 1/min. In contrast, all mutants started with a 10-fold higher EB load, which in about 50% of those cells decreased to the same level as in the wild-type after 10 min. However, even after 40 min a large proportion of mutant cells were still carrying high loads of EB, which was most pronounced for the *mexB::*Km and *oprM::*Km mutants (Figure 6). This indicated that indeed EB-efflux was impaired in the mutants, and suggested that 2-HBP efflux could be disrupted as well.

### 2-HBP transport in wild-type and mutant HBP1

**Figure 7:**
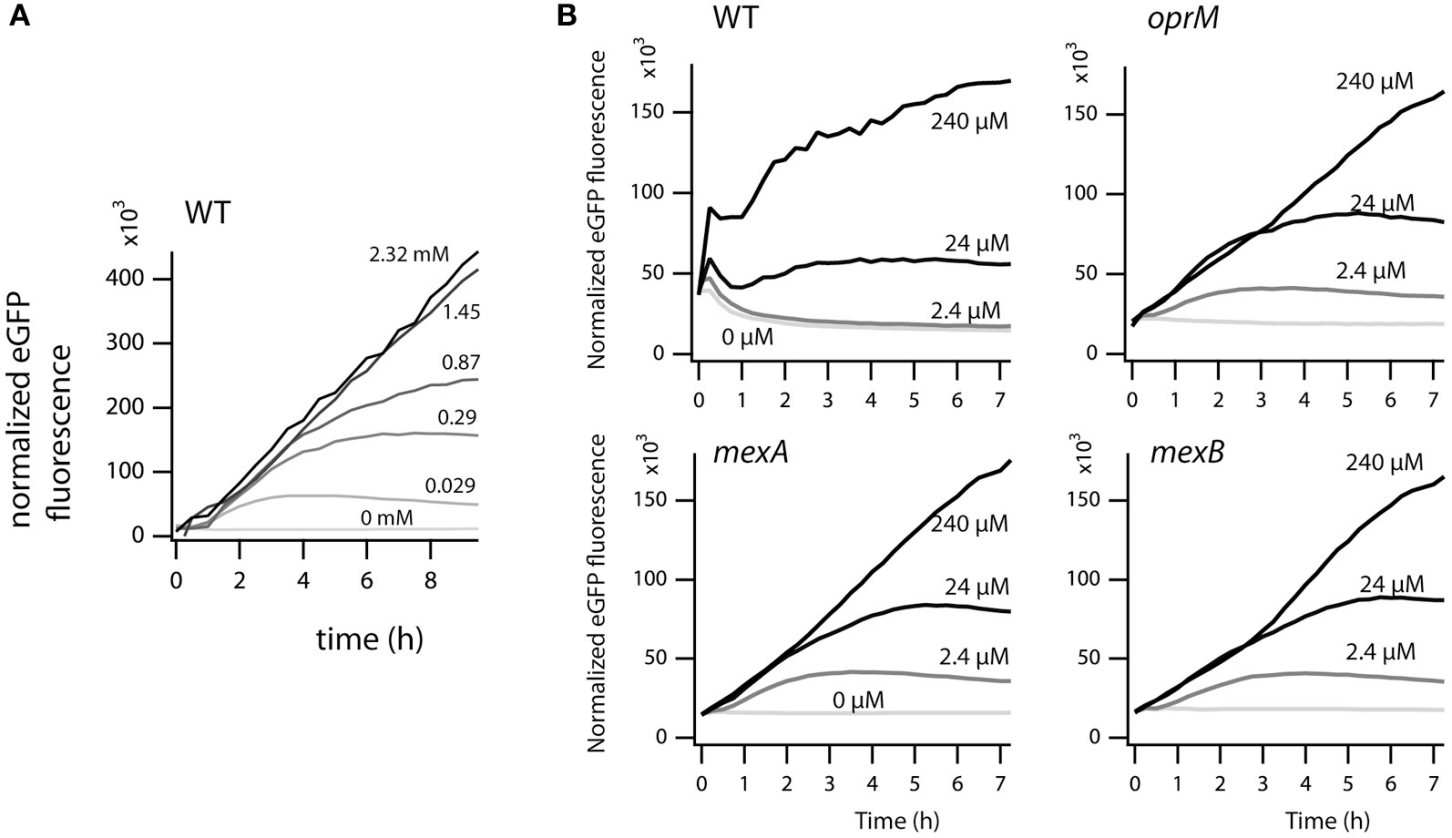
**Effect of external 2-HBP concentration on induction of GFP over time from the 2-HBP and HbpR-dependent P_C_-promoter. (A)** *P. azelaica* HBP1 wild type containing the reporter plasmid pME6012_hbpR_gfp exposed to 2-HBP concentrations between 0 and 2.3 mM. **(B)** *P. azelaica* HBP1 wild-type compared to *mexB*::Km, *mexA::*Km and *oprM::*Km insertion mutants transformed with pME6012_hbpR_gfp exposed or not to 2-HBP at 2.4, 24, or 240 μM. The GFP fluorescence signal is normalized by the culture density at every time point.

In order to measure the effect of the *mexA, mexB*, or *oprM* interruptions in *P. azelaica* HBP1 on 2-HBP transport (*here*: the combined effect of in- and efflux), we used an indirect assay since radio-active 2-HBP was not available for direct measurement of uptake or efflux. The derivative assay is based on the intracellular measurement of 2-HBP by the HbpR protein, which controls expression of the GFP reporter protein from the P_c_-promoter (Jaspers et al., 2000; Beggah et al., 2008). Previous results from such a 2-HBP-responsive *E. coli* bioreporter indicated that 2-HBP likely enters the cell by spontaneous partitioning and diffusion through the cellular membranes (Beggah et al., 2008). GFP fluorescence is thus an indirect (delayed) measure of the intracellular 2-HBP concentration. Wild-type *P. azelaica* and the three mutant strains were equipped with a plasmid carrying the *hbpR* gene under control of its own promoter and the GFP gene under control of the HbpR-dependent and 2-HBP inducible P_c_-promoter (Jaspers et al., 2000; Beggah et al., 2008). Indeed, GFP expression was induced in a 2-HBP-dependent manner in wild-type HBP1 (Figure 7A). Interestingly, at 2-HBP concentrations of 0.029, 0.29, and 0.87 mM GFP levels increased in the first period after induction but then leveled off. This suggested that the cells had metabolized all the 2-HBP and no further induction occurred. At higher 2-HBP outside concentrations the cells use longer to metabolize all 2-HBP and, consequently, the GFP induction process continues (Figure 7A). In contrast, the mutant strains were much more sensitive to 2-HBP than wild-type HBP1 (Figure 7B). GFP expression was already induced at 2.4 μM 2-HBP, indicating that it enters the cells but is not expelled as effectively as in the wild-type (Figure 7B). GFP values saturated at 2.4 and 24 μM 2-HBP concentration but not at 0.24 mM (Figure 7B), further suggesting that low 2-HBP concentrations can be metabolized by the mutant cells, but that metabolism stops at higher (0.24 mM) 2-HBP concentrations.

## Discussion

Here we analyzed the basis for the tolerance against 2-HBP toxicity in *P. azelaica* HBP1, which is one of the few reported strains to grow on 2-HBP as sole carbon and energy source (Kohler et al., 1988). By screening a large library of transposon mutants of HBP1 for absence of growth on 2.7 mM 2-HBP we recovered a majority of 2-HBP growth-defective mutants with insertions in three genes encoding proteins with high similarity to known efflux systems of the MexAB-OprM type of *P. aeruginosa* (Li et al., 1998) (Table S1, Data File S1). This suggested that constant efflux of 2-HBP is absolutely essential for maintaining productive metabolism and cellular growth, because of its toxicity. Further analysis of the possible toxic action using physiological dyes indicated that external concentrations above 0.5 mM 2-HBP cause wild-type cells to arrest growth as in stationary phase (given the behavior upon SYTO9/EB staining, Figure 3) and results in membrane potential uncoupling similar to treatment with sodium azide (given results of DiOC_2_(3) staining, Figure 4). Membrane potential uncoupling could be due to the propensity of 2-HBP to partition in and out the membrane while acting as proton shuttle (pKa = 9.4 at 25°C; http://sitem.herts.ac.uk/aeru/footprint/eu/Reports/1340.htm) (Escher et al., 2001).

Indeed, mutants with interrupted *mexA, mexB*, or *oprM* genes did not grow at such high (above 0.5 mM) 2-HBP concentrations, but they do at much lower concentrations (5–100 μM), albeit with reduced growth rates compared to HBP1 wild-type (Figure 2). Together, these results demonstrated that 2-HBP is toxic to *P. azelaica* even in the low μM-range, but suggested that the activity of a multidrug efflux system is needed to alleviate the toxic action by constantly removing 2-HBP from the intracellular environment to low levels. We could confirm this hypothesis by using a 2-HBP inducible gene reporter system introduced into HBP1 wild-type and mutants. Mutants but not wild-type showed activation of the gene reporter even at an outside concentration of 2.4 μM 2-HBP (Figure 7), indicating that 2-HBP is entering the cells but is not expelled. Previous work with a similar 2-HBP-responsive gene reporter in *E. coli* showed that induction already occurs at 0.5 μM 2-HBP (Beggah et al., 2008). This indicates that the main transcription activator for induction of 2-HBP metabolism (HbpR) will start to induce expression of the *hbpCAD* genes, which, in a matter of minutes will lead to enzyme production and disappearance of 2-HBP (Jaspers et al., 2000, 2001). GFP expression in the *mexA, mexB* or *oprM* mutants leveled off at 2.4 and 24 μM 2-HBP outside concentrations, suggesting that cells still induce and carry out 2-HBP metabolism, causing it to disappear from the intracellular environment, upon which further GFP induction from the reporter stalls. At higher concentrations (0.24 mM), however, GFP induction in the mutant strains continues, from which we conclude that the cells arrest 2-HBP metabolism. In contrast, GFP expression in wild-type cells still levels off at 0.87 mM 2-HBP (Figure 7B). Hence, 2-HBP metabolism is still active and cells only measurably show signs of toxicity above this range (Figures 3, 4) but continue to metabolize 2-HBP up to the water solubility level (2.7 mM). The exact reason as to why mutant cells also seem to stop metabolizing 2-HBP at concentrations of 0.24 mM is not immediately clear, but could relate to the interruption of cellular respiration and proton gradient uncoupling as evidenced from the DiOC_2_(3) staining (Figure 4).

Assays using EB as substrate indicated that the *P. azelaica* MexAB-OprM proteins form a typical multidrug efflux system, which, in analogy, likely expels antimicrobial compounds other than 2-HBP from the cell if necessary. Interestingly, several studies have shown that tolerance of e.g., *P. aeruginosa* to the widely used disinfectant triclosan is also mediated by the MexAB-OprM system (and further paralogs) (Chuanchuen et al., 2002, 2003; Mima et al., 2007). Triclosan (5-chloro-2-(2,4-dichlorophenoxy)-phenol) is a similar compound as 2-HBP, and the MexAB-OprM efflux pump in *P. aeruginosa* confers resistance up to the maximum aqueous solubility of triclosan (128 mg/L, 0.4 mM) in solution or up to 1024 mg/L in case of plates with organic solvents, while decreasing to 16 mg/L (5.5 μM) in absence of MexAB-OprM (Chuanchuen et al., 2003). A variety of mutations can be selected for which—in absence of a proper functioning MexAB-OprM pump, change expression of paralog RND pumps and confer additional tolerance to e.g., triclosan (Mima et al., 2007). Similarly, overexpression of the RND-type efflux pump AcrAB was responsible for increased tolerance to triclosane (McMurry et al., 1998; Levy, 2002; Braoudaki and Hilton, 2004, 2005). In this respect it is interesting to note that expression of *mexAB-oprM* in *P. azelaica* seemed constitutive and not dependent on 2-HBP exposure (Czechowska, unpublished). A few independent transposon mutants were found in a gene cluster putatively coding for an ABC-type transport system (Figure 1), suggesting it may have an additional role in preventing 2-HBP toxicity. One such system encoded by the *linKLMN* genes has been previously implicated in protecting against lindane toxicity in *Sphingobium japonicum* (Endo et al., 2007).

The MexAB-OprM system of *P. azelaica* thus adds to the impressing capacity of this efflux pump type to confer tolerance to solvents or antimicrobial compounds (Ramos et al., 2002; Alvarez-Ortega et al., 2013), but in case of strain HBP1 in particular, also demonstrates how recruitment of an effective efflux system is essential for a metabolic pathway to operate productively. Given the increased importance of biodesulfurization processes to clean up oil and coal (Alves and Paixao, 2011; Gunam et al., 2013), strains such as *P. azelaica* HBP1 should find increased usage to decontaminate the produced 2-HBP, before this is released in an untreated form and can do further environmental damage because of its toxicity.

### Conflict of interest statement

The authors declare that the research was conducted in the absence of any commercial or financial relationships that could be construed as a potential conflict of interest.

## Supplementary material

Figure S1 **Membrane damage in *P. azelaica* HBP1 wild-type cells as a function of exposure to 2-HBP**.

Figure S2 **Exponentially growing cells of *Pseudomonas azelaica* HBP1 on 5 mM sodium succinate exposed or not to CCCP (15 μM), sodium azide (4 g per L), or valinomycin (1 μM) over 1 (A) and 3 h (B) stained with membrane potential dye DiOC2(3)**.

Table S1 **Insertion positions of transposons in the *mexA-oprM* and genes for putative toluene resistance protein regions of *Pseudomonas azelaica* HBP1, that cause cells to abolish growth in the presence of 2.7 mM 2-hydroxybiphenyl**.

Data File S1 **DNA sequences and deduced amino acid sequences of the *mexA-oprM* region of *Pseudomonas azelaica* HBP1 and the region with genes encoding a putative toluene resistance protein**. Nucleotide numbering according to a draft genome assembly of *Pseudomonas azelaica* HBP1 (submission in preparation), corresponding to Table S1 and Figure 1.
