# Supplementary material for: Characterization of a MexAB-OprM efflux system necessary for productive metabolism of Pseudomonas azelaica HBP1 on 2-hydroxybiphenyl
Source: Front Microbiol. 2013 Jul 19;4:203. doi: 10.3389/fmicb.2013.00203 (PMC3715732; doi:10.3389/fmicb.2013.00203)
Supplement: Figure S1 — Membrane damage in P. azelaica HBP1 wild-type cells as a function of exposure to 2-HBP. [file DataSheet1.DOCX]

**Characterization of a MexAB-OprM Efflux System Necessary for Productive Metabolism of *Pseudomonas azelaica* HBP1 on 2-Hydroxybiphenyl.**

Czechowska K., C. Reimmann and J. R. van der Meer*

**Department of Fundamental Microbiology**

**University of Lausanne**

**Bâtiment Biophore**

**Quartier UNIL-Sorge**

**1015 Lausanne, Switzerland**

**Tel. +41 21 6925630**

**Email: janroelof.vandermeer@unil.ch**

*** corresponding author**

**Figure S1**. Membrane damage in *P. azelaica* HBP1 wild-type cells as a function of exposure to 2-HBP

**Figure S2**. Exponentially growing cells of *Pseudomonas azelaica* HBP1 on 5 mM sodium succinate exposed or not to CCCP (15 µM), sodium azide (4 g per L), or valinomycin (1 µM) over 1 (A) and 3 h (B) stained with membrane potential dye DiOC2(3).


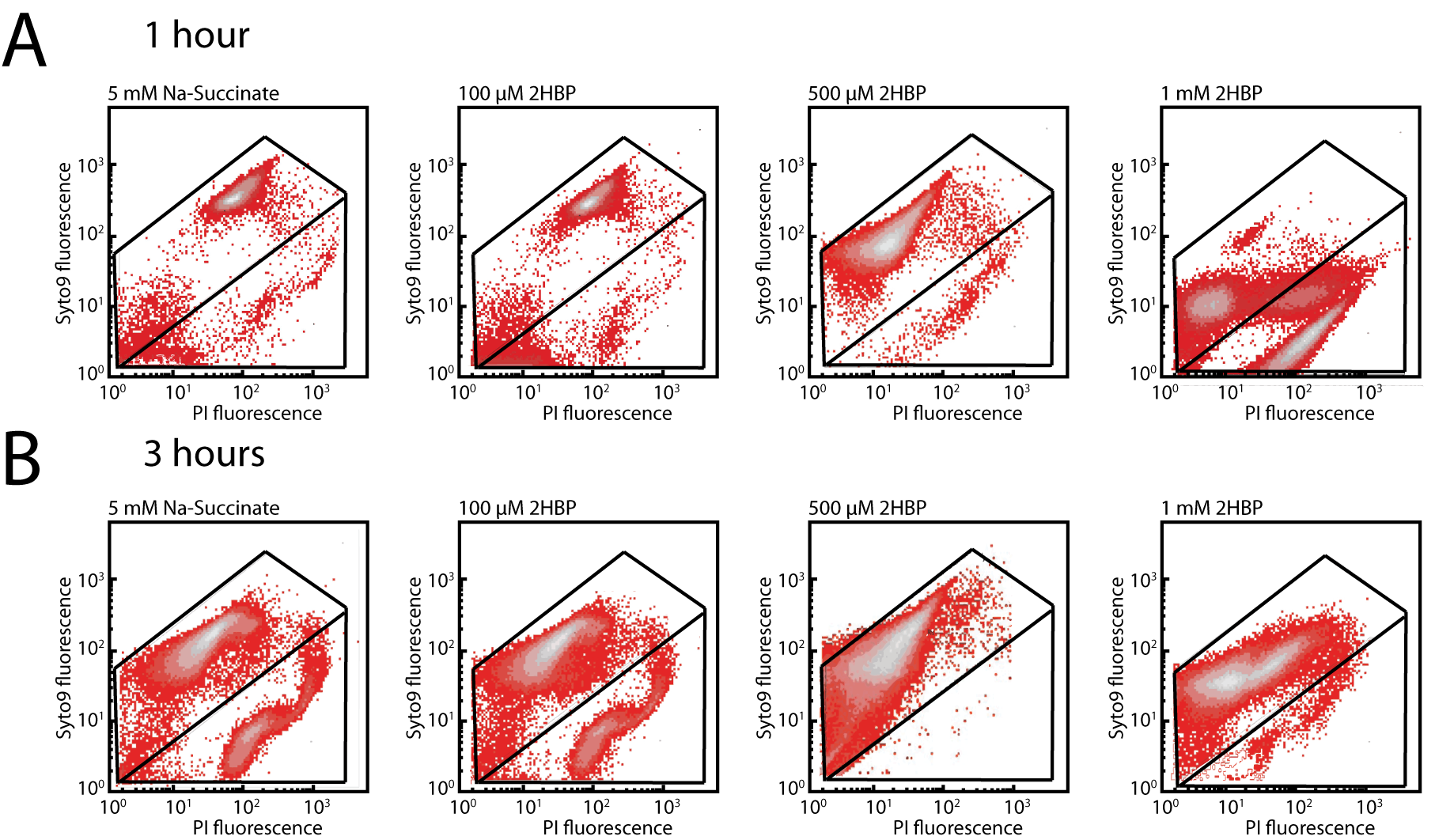


Figure S1. Membrane damage in *P. azelaica* HBP1 wild-type cells as a function of exposure to 2-HBP. A) Exponentially growing cells on 5 mM sodium succinate exposed or not to 2-HBP at 100 µM, 0.5 and 1 mM concentrations. Cells sampled after 1 h exposure and stained with SYTO9 plus Propidium Iodide (PI). B) as for A, but sampled and stained after 3 h exposure.


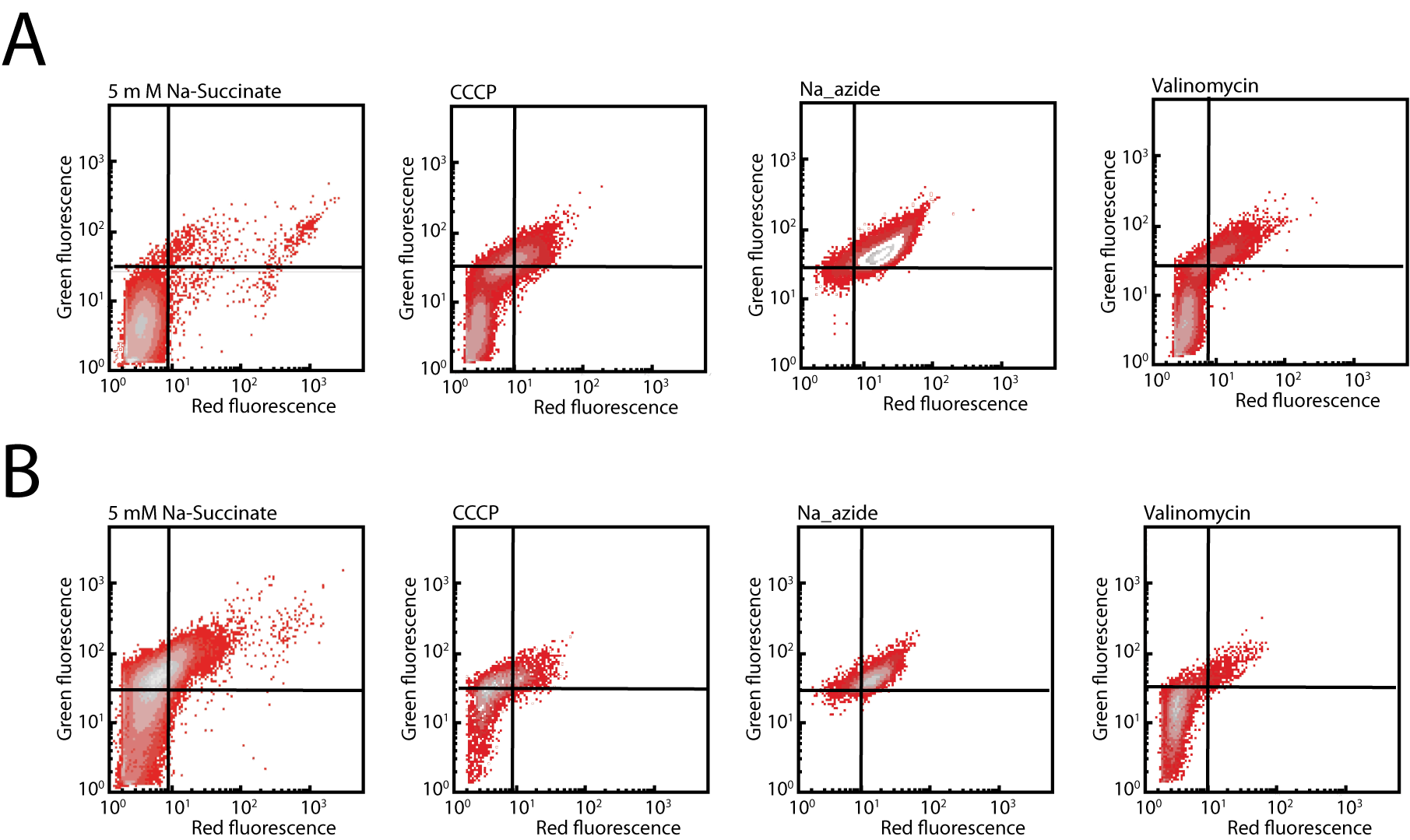


Figure S2. Exponentially growing cells of *Pseudomonas azelaica* HBP1 on 5 mM sodium succinate exposed or not to CCCP (15 µM), sodium azide (4 g per L), or valinomycin (1 µM) over 1 (A) and 3 h (B) stained with membrane potential dye DiOC2(3). Quadrants were set on the basis of non-exposed cells and cells treated with sodium azide.
