# Supplementary material for: Characterization of a MexAB-OprM efflux system necessary for productive metabolism of Pseudomonas azelaica HBP1 on 2-hydroxybiphenyl
Source: Front Microbiol. 2013 Jul 19;4:203. doi: 10.3389/fmicb.2013.00203 (PMC3715732; doi:10.3389/fmicb.2013.00203)
Supplement: Table S1 — Insertion positions of transposons in the mexA-oprM and genes for putative toluene resistance protein regions of Pseudomonas azelaica HBP1, that cause cells to abolish growth in the presence of 2.7 mM 2-hydroxybiphenyl. [file DataSheet2.DOCX]

Table S1. Insertion positions of transposons in the *mexA-oprM* and genes for putative toluene resistance protein regions of *Pseudomonas azelaica* HBP1, that cause cells to abolish growth in the presence of 2.7 mM 2-hydroxybiphenyl.

| **Insertion position** | **Designation** | **Gene name** | **Gene product** |
| --- | --- | --- | --- |
| 4681647 | PA_4562 | *mexA* | Multidrug resistance protein MexA |
| 4681702 |  | *mexA* |  |
| 4681706 |  | *mexA* |  |
| 4681714 |  | *mexA* |  |
| 4681772 ^a^ |  | *mexA* |  |
| 4681772 ^a^ |  | *mexA* |  |
| 4681811 |  | *mexA* |  |
| 4681898^a^ |  | *mexA* |  |
| 4681898 ^a^ |  | *mexA* |  |
| 4682075 |  | *mexA* |  |
| 4682152 |  | *mexA* |  |
| 4682540 |  | *mexA* |  |
| 4682669 | PA_4563 | *mexB* | Multidrug resistance protein MexB |
| 4682810 ^a^ |  | *mexB* |  |
| 4682810 ^a^ |  | *mexB* |  |
| 4683315 |  | *mexB* |  |
| 4683482 |  | *mexB* |  |
| 4684209 |  | *mexB* |  |
| 4684368 |  | *mexB* |  |
| 4684481 |  | *mexB* |  |
| 4684514 |  | *mexB* |  |
| 4684520 |  | *mexB* |  |
| 4684582 |  | *mexB* |  |
| 4684628 |  | *mexB* |  |
| 4684816 |  | *mexB* |  |
| 4685149 |  | *mexB* |  |
| 4685876 | PA_4564 | *oprM* | Outer membrane protein OprM |
| 4685890 ^a^ |  | *oprM* |  |
| 4685890 ^a^ |  | *oprM* |  |
| 4685890 ^a^ |  | *oprM* |  |
| 4686301 |  | *oprM* |  |
| 4686475 |  | *oprM* |  |
| 4686881 |  | *oprM* |  |
| 4687062 |  | *oprM* |  |
| 4687111 |  | *oprM* |  |
| 5380855 | PA_5195 |  | putative ABC transporter ATP binding protein |
| 5382362 | PA_5197 |  | putative toluene tolerance ABC efflux transporter, periplasmic component |
| 5382757 | PA_5198 |  | putative toluene tolerance ABC efflux transporter, auxilliary component |
| 5382889 | PA_5198 |  |  |
| 5383188 | PA_5199 |  | putative anti-anti-sigma regulatory factor |
| 5383192 | PA_5199 |  |  |

a) Recovered from independently generated transposon libraries

Position numbering according to a draft assembled genome sequence of *Pseudomonas azelaica* HBP1 (submission in preparation).
