# Supplementary material for: Characterization of a MexAB-OprM efflux system necessary for productive metabolism of Pseudomonas azelaica HBP1 on 2-hydroxybiphenyl
Source: Front Microbiol. 2013 Jul 19;4:203. doi: 10.3389/fmicb.2013.00203 (PMC3715732; doi:10.3389/fmicb.2013.00203)
Supplement: Data File S1 — DNA sequences and deduced amino acid sequences of the mexA-oprM region of Pseudomonas azelaica HBP1 and the region with genes encoding a putative toluene resistance protein. Nucleotide numbering according to a draft genome assembly of Pseudomonas azelaica HBP1 (submission in preparation), corresponding to Table S1 and Figure 1. [file DataSheet3.DOCX]

**Characterization of a MexAB-OprM Efflux System Necessary for Productive Metabolism of *Pseudomonas azelaica* HBP1 on 2-Hydroxybiphenyl.**

Czechowska K., C. Reimmann and J. R. van der Meer*

**Department of Fundamental Microbiology**

**University of Lausanne**

**Bâtiment Biophore**

**Quartier UNIL-Sorge**

**1015 Lausanne, Switzerland**

**Tel. +41 21 6925630**

**Email: janroelof.vandermeer@unil.ch**

**Data File S1**: DNA sequences and deduced amino acid sequences of the *mexA-oprM* region of *Pseudomonas azelaica* HBP1 and the region with genes encoding a putative toluene resistance protein.

Nucleotide numbering according to a draft genome assembly of *Pseudomonas azelaica* HBP1 (submission in preparation), corresponding to Table S1 and Figure 1.

LOCUS mexR_oprM 7644 bp DNA linear 10-JUN-2013

FEATURES Location/Qualifiers

source 4679634..4687277

/dnas_title="mexR_oprM region of Pseudomonas azelaica"

misc_feature complement(4680765..4681241)

/note="mexR"

/translation="MVELINLIYTRFTVTEPMTYTLNPDLPQTLMKVLDHLRESLQDE

LCAEGIDLTPPDIRLLELIGADAGQSLGRKMCRDKALVTRKIREMETLGLVRRERNPD

DQRSFQLFLTEAGSRIDERTQAILARTHDSLFAPLDDEEQRTLTQLLRQCLQGRGD"

misc_feature 4681461..4682618

/note="mexA"

/translation="MQRKPAMRALVPALLVAMATLAGCDKAQAPQEKQIPEVGVVTLQ

PQEVTLSTELPGRTTAYRIAEVRPQVNGIILKRLFKEGSDVKEGQQLYQIDPATYDAT

LQSAQANLVSTQQQAERYKELVKDEAVSKQQYADAQAAYLQAKATVDTAKINVRYTKV

YSPISGRIGRSSVTEGALVQNGQATALATVQQLDPIYVDVTQSSTALIRLRREMAAGQ

LEKAGDNAAKVKLYLEDGSEYPIEGKLEFSEVSVDQGTGSVTVRAIFPNPKKELLPGM

FVHAQLEEGIKKQAILAPQQGVTRDFRGMATALVLNGEDKVELRTIKAERTVGAYWLV

SDGLKPGDRLITEGLQYVQPGAQAKAVPAKNVAPTAGAADKPAAAEPAKQG"

misc_feature 4682634..4685777

/note="mexB"

/translation="MSKFFIDRPIFAWVIALVIMLAGGLSILKLPVNQYPAIAPPAIA

IQVSYPGASAETVQDTVVQVIEQQMNGLDHLRYISSESNSDGSMTITVTFDQGTSPDI

AQVQVQNKLQLATPLLPQEVQQQGIRVTKAVKNFLMVVGVVSEDGSMTKEDLSNYIVS

NIQDPLSRTAGVGDFQVFGSQYAMRIWLDPAKLNSFQMTPGDVKTAIQAQNVQISSGQ

LGGLPAVKGQQLNATIIGKTRLQSAEQFKEILLKVNADGSQVRLKDVADVALGGQDYS

INAQFNGKPASGIAIKLATGANALDTAKSIRATLATLEPFFPQGMKIVYPYDTTPVVS

ASIHEVVKTLGEAILLVFLVMYLFLQNFRATLIPTIAVPVVLLGTFGVLAAFGFSINT

LTMFGMVLAIGLLVDDAIVVVENVERVMAEEGLSPREAARKSMGQIQGALVGIAMVLS

AVFLPMAFFGGSTGVIYRQFSITIVSAMALSVIVALILTPALCATMLKPIEKGDHGEH

KRGFFGWFNRKFLATTHGYERGVTSILNHRMPYLLMYVLILGGMVYLFTRIPTAFLPD

EDQGVLFAQVQTPAGSSAERTQVVVDSMREYLLEKESSSVASVFTVTGFNFAGRGQSS

GMAFIMLKPWEERPGAENSVFALAQRAQMHFFSFKDAMVFAFAPPAVLELGNAVGFDI

FLQDQAGVGHEVLMQARNQFLGMASQNPALQRVRPNGLNDEPQYKLLIDDEKASALGV

SLADINTTVSIAWGSNYVNDFIDRGRVKKVYLQGRPNSRMSPEDLDKWYVRNSSGEMV

PFSAFATGEWSYGSPKLARYNGVPAMEVLGEPAPGRSTGEAMAAVEEMVKQLPKGVGY

SWTGLSYEERLSGSQAPALYALSLIVVFLCLAALYESWSIPFSVMLVVPLGVIGALLA

TSMRGLSNDVFFQVGLLTTIGLSAKNAILIVEFAKELHEREGKPIVAAAIEACRMRLR

PIVMTSLAFVLGVLPLAISTGAGSGSQHAIGTGVIGGMITATILAIFWVPLFYVVVST

LFKDKLSKEQEATEKGH"

misc_feature 4685707..4687248

/note="oprM"

/translation="MRKSLLLPSLHLAIAAGVLSGCSLIPDYERPEAPVAAAYPQGDA

YDAAQPANAGADIGWREFFKDPQLQRLVEVSLENNRDLRVAALNIEAYRAQYRIQRAD

LFPAVDASFDGSRQRLPADLSQTGSSMISSQYGATLGITSWEIDLFGRLRALRDQALE

QYFATEEARRSTQTSLVANVANAYLTLRADQAQLQLTRDTLGTYQKSYDLTKRSYDVG

VASALDLRQAQTSVESARATLAQYTRLVAQDQNALVLLLGSGLPADLPQGRGLGDELL

ATVPAGLPSDLLQRRPDILQAEHKLKAANANIGAARAAFFPNVSLTANAGTISPDLGG

LFDGGSGTWLFKPSISLPIFNAGSLRASLDLAKVQKDINVAQYEKAIQTAFSEVADGL

AARGTFNEQLQAQRALVEASSEYYRLADKRYRTGVDNYLTLLDAQRSLFSAQQQLISD

RLNQLTSEVNLYKALGGGWQATAAQAKPISSEAPEGRLF"

ORIGIN

4679634 gcctgttcgg cctgggcctg tggatgcgtg agctggacta ctacgacacc tggtaccacc

4679694 gcgccccgga aatccacaaa agcatcggca tcctgctggc catcgcactg atcgtgcgtg

4679754 tgctctggcg cttcatcagc ccgtcgccgc cgaccccgtc caaccacggc aatctcaccc

4679814 gcctggccac caagctcggc cacctggcgc tctacgcgct gctgttcgcc gtgatcatcg

4679874 ccggctacct gatctccacc gccgaaggca aacccatcag cgtgttcggc tggttcgacg

4679934 tgcccgccac actgagcggt atcaccgacc aggccgacat cgccggtgcg atccacctgt

4679994 acctggcctg ggccctggtc gtcctggccg tgctgcacgc cctggcggcg ttcaaacacc

4680054 atttcctcga ccgcgatgcc acgctggtgc gcatgctcgg tcgtgctgcg aaataacacc

4680114 tcaccccaaa gggagattgc ttcaatgctg aagaagacgt tcgccgcgct ggcgctgggt

4680174 accgcactgt tctccgctgg ccaggccatg gccgcagact acaagatcga caaggaaggc

4680234 cagcatgcct tcatcgaatt ccgcatcaag cacctgggtt acagctggct gtacggccgc

4680294 ttcgacgatt tcgacggtgc cttcaccttc gacgagaaga acccgtccgc cgacaaggtc

4680354 aaggtgacca tcaacaccaa cagcgtgaac tccaaccacg ccgagcgtga caagcacctg

4680414 cgcagcccgg acttcctcaa cgtctcgaag aacccgactg caaccttcga atccaccgcc

4680474 gtgaaggctg atggcaagaa tgccgagatc accggcaacc tgaccctcaa tggcgtgacc

4680534 aagccggtca ccatcaaggc tgaactgatc ggccagggcg atgatccgtg gggcggctac

4680594 cgcgccggct tcctcggcac caccaccctc aagctgaagg acttcaatat ccagcgcgac

4680654 ctcggcccgg cttcccagga agtcgagctg accctgtcgg tcgaaggggt gcgtcagtaa

4680714 gacgcaggac tgcgaaaacg ccggcgaatg ccggcgtttt cgtttgtggc cttagtctcc

4680774 ccggccttgc aggcattgcc gcagaagctg ggtgagggtg cgctgttctt cgtcgtccag

4680834 cggcgcgaac agactgtcat gggttcgtgc gaggatggcc tgggtgcgct cgtcgatgcg

4680894 gctcccggcc tcggtgagga acagctggaa gctgcgctga tcgtccgggt tgcgctcgcg

4680954 gcgaaccagg ccgagcgtct ccatttcacg gatcttgcgg gtcaccagcg ccttgtcgcg

4681014 gcacatcttg cgccccaggc tctggcccgc atcggcgccg atcagctcga gaaggcggat

4681074 gtccggcggg gtgaggtcga tgccctcggc gcacagttcg tcctggaggc tctcacgcaa

4681134 atgatcgagc accttcatca gggtctgtgg caggtcggga ttcagggtat aggtcatcgg

4681194 ctcggtcacg gtaaaccttg tgtaaatcag gttgatcagc tcaaccattt tttcgtattt

4681254 tagttgatgt tatcaacttt gttcgtcgcc gcgactgcct tgcgggaaac ctactgcgat

4681314 atcggccttt ggctaaaacg tttcagtagt tttccctcag ggctttcgga cgtttacaaa

4681374 cacagctgaa tgtaagtatt tttgccagct tttccggacc ggcaagccca cagccaaacc

4681434 ggatgctttg ctcacgagga caacgatatg caacgaaagc cagccatgcg cgccctggtc

4681494 ccggccctgc tcgtcgccat ggccactctt gccggctgcg acaaagccca ggccccgcag

4681554 gagaagcaga tccccgaagt cggtgtggta accctgcagc cgcaggaagt gaccctgagc

4681614 accgaactgc cgggccgcac cactgcgtat cgcatcgccg aagttcgccc gcaggtgaac

4681674 ggcatcatcc tcaagcgcct gttcaaggaa ggcagcgacg tcaaggaagg ccagcagctg

4681734 taccagatcg acccggccac ctatgacgct accctgcaga gcgcccaggc caacctggtg

4681794 tccacccagc agcaggccga gcgctacaag gaactggtga aagacgaggc ggtgagcaag

4681854 cagcaatacg ccgacgccca ggccgcctat ctgcaggcca aggccaccgt cgacaccgcc

4681914 aagatcaacg tgcgctacac caaggtgtat tcgccgattt ccggccgcat cggccgctcc

4681974 agcgtgaccg agggcgccct ggtgcagaac ggtcaggcca ccgcgctggc caccgtgcag

4682034 caactggacc cgatctacgt cgacgtcacg cagtcttcca ccgcgctgat ccgcctgcgc

4682094 cgcgagatgg ccgccgggca actggagaag gccggcgaca atgccgccaa ggtcaagctg

4682154 tacctggaag acggctccga ataccccatc gagggcaagc tggagttctc cgaggtttcg

4682214 gtcgaccagg gcaccggctc ggtgaccgtg cgcgccatct tccccaaccc gaagaaggaa

4682274 ctgctgccgg gcatgttcgt ccatgcgcag ctggaggagg gcatcaagaa gcaggccatc

4682334 ctcgcgccgc agcagggcgt cacccgtgac ttccgtggca tggcgactgc cctggtgctc

4682394 aatggcgaag acaaggtcga gctgcgcacc atcaaggccg agcgcaccgt tggcgcctac

4682454 tggctggtca gcgatggcct caagcccggc gaccgcctga tcaccgaagg cctgcagtat

4682514 gtccagccgg gcgcccaggc caaggccgta cccgcgaaga acgtggcgcc caccgctggt

4682574 gccgccgaca agcccgccgc cgctgaaccg gccaagcagg gttaatcaag gggattcgtc

4682634 atgtcgaagt ttttcattga ccggcccatt ttcgcctggg taatcgccct ggtgatcatg

4682694 ctggcgggcg gtctgtcgat cctcaagttg ccggtgaacc agtacccggc tatcgccccg

4682754 ccggccatcg ccatccaggt gagctacccg ggcgcctccg ccgagacggt gcaggacacc

4682814 gtggtgcagg tgatcgagca gcagatgaac ggtctcgacc atctgcgcta catctcctcg

4682874 gagtccaact ccgacggcag catgaccatc accgtgacct tcgaccaggg caccagccct

4682934 gacatcgccc aggtccaggt gcagaacaag ttgcagctgg ccaccccgct cctgccgcag

4682994 gaagtacagc agcagggcat ccgcgtgacc aaggcggtga agaacttcct gatggtggtc

4683054 ggcgtcgtct ccgaagacgg cagcatgacc aaggaagacc tgtcgaacta catcgtctcc

4683114 aacatccagg acccgctgtc gcggactgcc ggtgtgggcg acttccaggt gttcggttcg

4683174 cagtacgcca tgcgtatctg gctggacccg gcgaagctca acagcttcca gatgactccg

4683234 ggcgacgtga agacggcgat ccaggcgcag aacgtgcaga tttcctccgg ccagctgggc

4683294 ggcctgcctg cggtcaaggg ccagcagctc aacgccacga tcatcggcaa gacccgcctg

4683354 cagagcgccg agcagttcaa ggaaatcctc ctcaaggtca acgccgacgg ttcccaggtt

4683414 cgcctgaagg acgttgccga cgtggccctg ggcggccagg actacagcat caacgcccag

4683474 ttcaacggca agccggcctc cggtatcgcg atcaagctgg ccaccggcgc caacgcgctg

4683534 gacaccgcca agtcgatccg cgccaccctg gccacgctgg agccgttctt cccgcagggc

4683594 atgaagatcg tctaccccta cgacaccacc ccggtggtct ccgcctccat ccatgaggtg

4683654 gtgaagaccc tgggcgaagc gatcctgctg gtgttcctgg tgatgtacct gttcctgcag

4683714 aacttccgcg ccacgctgat cccgacgatc gccgtgccgg tggtcctgtt gggcaccttc

4683774 ggcgtactcg ccgccttcgg cttctcgatc aacaccctga ccatgttcgg catggtgctg

4683834 gcgatcggcc tgttggtgga cgatgccatc gtcgtggtgg aaaacgtcga gcgggtgatg

4683894 gccgaggagg gcctgtcgcc ccgcgaggcg gcgcgcaagt ccatgggcca gatccagggc

4683954 gcgctggtgg gtatcgccat ggtgctgtcg gcggtgttcc tgccgatggc cttcttcggc

4684014 ggctccaccg gggtgatcta ccggcagttc tccatcacca tcgtctcggc catggccctg

4684074 tcggtgatcg tggcgctgat cctcaccccg gcgctctgcg ccaccatgct caagcccatc

4684134 gagaagggcg accatggcga gcacaagcgc ggcttcttcg gctggttcaa ccgcaagttc

4684194 ctcgccacca cccatggcta cgagcgcggc gtcacctcga tcctcaatca ccgcatgccg

4684254 tacctgctga tgtacgtgct gatcctcggc ggcatggtct acctgttcac ccgcattccc

4684314 acggcgttcc tccccgacga ggaccagggc gtgctgttcg cccaggtgca gaccccggcc

4684374 ggttcgtccg ccgagcgcac ccaggtggtg gtggactcga tgcgcgagta cctgctggag

4684434 aaggaaagca gctcggtggc ctcggtgttc accgtgaccg gcttcaactt cgccggccgt

4684494 ggccagagct cgggcatggc gttcatcatg ctcaagccct gggaagagcg tccgggcgcg

4684554 gagaacagcg tgttcgcgct ggcccagcgt gcgcagatgc acttcttcag cttcaaggat

4684614 gcgatggtgt tcgccttcgc cccgccggcg gtactcgaac tgggtaacgc cgtgggcttc

4684674 gacatcttcc tccaggacca ggctggcgtc ggccatgaag tgctgatgca ggcgcgcaac

4684734 cagttcctcg gcatggcctc gcagaaccct gctctgcagc gcgtgcgtcc caacggcctg

4684794 aacgacgagc cgcagtacaa gctgctgatc gacgacgaga aggccagtgc gctgggcgtc

4684854 tcgctggcgg acatcaacac caccgtgtcg attgcctggg gctcgaacta cgtcaacgac

4684914 ttcatcgacc gcggtcgggt gaagaaggtc tacctgcaag gccgcccgaa ttcgcggatg

4684974 agcccggaag acctggacaa gtggtacgtg cgcaacagct ccggggagat ggtgccgttc

4685034 agcgccttcg ccaccggcga atggagctat ggctcgccga agctggcgcg ctacaacggc

4685094 gtcccggcca tggaagtcct cggtgagccg gccccgggcc gctccactgg tgaggcgatg

4685154 gccgcggtcg aggagatggt caagcagttg cccaagggcg tcggctactc ctggaccggt

4685214 ctgtcctacg aggaacgcct gtccggctcg caggctccgg cgctctatgc cctgtcgctg

4685274 atcgtggtgt tcctctgcct ggcggcattg tatgaaagct ggtcgatccc gttctcggtg

4685334 atgctggtgg tgccgctggg cgtcatcggt gcgctgctgg cgacgtccat gcgcggcctg

4685394 tccaacgacg tgttcttcca ggtgggcctg ttgacgacca tcggcctgtc ggcaaagaac

4685454 gcgatcctca tcgtggaatt cgccaaggag ctgcacgagc gcgagggcaa gcccatcgtc

4685514 gcggccgcca tcgaggcgtg ccggatgcgt ctgcgcccga tcgtcatgac ctccctggcg

4685574 ttcgtcctcg gcgtgttgcc gctggcgatc tccaccggtg caggctccgg cagccagcac

4685634 gcgatcggta ccggcgtgat cggcggcatg atcaccgcga ccatcctcgc gatcttctgg

4685694 gtaccgctgt tctatgtggt ggtcagtacc ctgttcaagg acaagctgtc caaggaacag

4685754 gaagccaccg agaaggggca ttgatatgag aaagtcctta ctgttgccct ctttgcattt

4685814 ggcgatagcg gccggtgtgt tgtccggctg ctcgctgatc cccgattacg agcgccccga

4685874 ggcgcccgtg gccgcggctt acccgcaggg cgatgcttac gatgcggcgc aaccggccaa

4685934 cgccggcgcc gacatcggct ggcgcgagtt cttcaaggac ccgcaactgc agcgactggt

4685994 cgaagtgtcg ctggaaaaca accgcgacct gcgcgtcgca gcgctgaaca tcgaagctta

4686054 ccgggcgcag taccgcatcc agcgggccga cctgttcccg gcggtggatg ccagcttcga

4686114 cggctcgcgc cagcgcttgc cggcggacct gtcgcagacc ggcagttcga tgatcagcag

4686174 ccagtacggt gcgaccctgg gcatcacgtc gtgggaaatc gacctgttcg gccgcctgcg

4686234 cgccctgcgt gatcaggcgc tggagcagta cttcgccacc gaagaggcgc gtcgcagcac

4686294 ccagaccagc ctggtggcca acgtcgccaa cgcctacctg acgctgcgcg ccgaccaggc

4686354 ccagttgcag ctgacccgcg acaccctcgg cacttaccag aagagctacg acctgaccaa

4686414 gcgcagctac gacgtcggtg tcgcatcggc gctggacctg cgccaggcgc agacctcggt

4686474 ggaaagcgca cgggcgactc tggcgcaata cacccgcctg gtggcgcagg accagaatgc

4686534 cctggtgctg ctgctcggtt ccggcctgcc ggcggacctg ccgcaaggcc gtggcctggg

4686594 tgacgaactg ctggccaccg tgccggcggg cctgccctcc gacctgctgc agcgccgccc

4686654 ggacatcctc caggcggagc acaagctcaa ggcggccaac gccaacatcg gcgcggcgcg

4686714 cgcggcgttc ttcccgaacg tcagcctgac cgccaacgcc ggcaccatca gcccggacct

4686774 cggtggcctg ttcgacggcg gttccggcac ctggctgttc aagccgtcga tcagcctgcc

4686834 gatcttcaac gccggcagcc tgcgcgcgag cctggacctg gccaaggtgc agaaggacat

4686894 caacgtcgcg cagtacgaga aagccattca gactgcgttc tccgaggtgg cggatggtct

4686954 tgccgcccgt ggcaccttca acgagcaact gcaggcgcag cgcgcgctgg tggaggccag

4687014 cagcgagtac taccgcctgg ccgacaagcg ctaccgcacc ggtgtggaca actacctcac

4687074 cctgctcgat gcgcaacgct cgctgttcag tgcccagcaa cagctgatct ccgaccgcct

4687134 caaccagctg accagcgagg tcaacctgta caaggccctc ggcggcggct ggcaggccac

4687194 ggcagctcag gccaagccga tctccagcga ggcccccgaa ggccggttgt tctgatcgag

4687254 aacagaaacg ccgccccttg gggc //

LOCUS PA5195_PA5200 4474 bp DNA linear 10-JUN-2013

FEATURES Location/Qualifiers

source 5382002..5384515

/dnas_title="PA5195_PA5200 region of Pseudomonas azelaica"

misc_feature 5382397..5383206

/note="PA_5195"

/product="toluene tolerance ABC efflux transporter, ATP-

binding protein"

/translation="MSTNDQYAVELKGLSFKRGTRKIFDNVDIRIPRGKVTGIMGPSG

CGKTTLLRLIAAQLRPASGEVWVNGQNLPTLGRSDLFDMRKQFGVLFQSGALFTDLDV

FENVAFPLRVHTELPEDMIRDIVLMKLQAVGLRGAVELMPDELSGGMKRRVALARAIA

LDPQILMYDEPFVGQDPIAMGVLVRLIRLLNDALGITSIVVSHDLAETASIADYIYIV

GDGRVLGHGTPADLQGLDDPRVRQFMKGIPDGPVPFHYPARDYRADLLGER"

misc_feature 5383206..5384003

/note="PA_5196"

/product="ABC-type transport system involved in resistance

to organic solvents, permease component"

/translation="MRKTSSLERVRLLGRAGLDVLESLGRSSLFLVHAIFGRRVHSGA

FQLLVKQLYSVGVLSLAIIVVSGLFIGMVLALQGYNILASYGSEQAVGQMVALTLLRE

LGPVVTGLLFAGRAGSALTAEIGNMKSTEQLSSLEMIGVDPLKYIIAPRLWAGFISMP

LLAAIFSVVGIWGGALVAVDWLGVYDGSYWSNMQNSVQFTKDILNGVIKSVVFAFVVT

WIAVFQGYDCEPTSEGISRATTRTVVYASLAVLGLDFILTALMFGDF"

misc_feature 5384003..5384476

/note="PA_5197"

/product="ABC-type transport system involved in resistance

to organic solvents, periplasmic component"

/translation="MQTRTLEIGVGLFILAGLLALLLLALRVSGLTIGNSGDTYKLYA

YFDNIAGVTVRGKVTMAGVTIGKVTAVDLDHDSYRGRVTMEINRQVNNLPDDSTASIL

TAGLLGEKYIGISVGGDEELLKDGDTIKDTQSALVLEDLIGKFLLNSVNKDDSKK"

misc_feature 5384488..5385135

/note="PA_5198"

/product="ABC-type transport system involved in resistance

to organic solvents, auxilliary component"

/translation="MLKTLRRGFLVLLAVLPLFAQAEQTAQQVVQQTVDSLLSDLKTN

KASYKANPQAFYDTLNRILGPVVDSEGIAKGVMTVKYSRQATPEQVKRFEETFKNSLF

QFYGNALLEYDNQDIRVLNSGKQEQDRASVNMEVVGSNGAVYPVQYTMVNQGGDWRLR

NVIINGINIGKLFRDQFADSMQKNRNDLEKTIAGWGLVVAKAKDAAKGQPEGSAE"

misc_feature 5385132..5385440

/note="PA_5199"

/product="putative anti-anti-sigma regulatory factor"

/translation="MSQGSITERSEGDLALAGVLDYRTGPALREQGRSLIAASKAASL

RLDCSAVEHSSSVGLSLLLSYTRDARKAGKTLQISGMPKDMQEIAKVGELLEILPLQH

"

misc_feature 5385551..5385790

/note="PA_5200 ttgF"

/product="predicted transcriptional regulator, BolA super-

family, ttgF"

/translation="MQAVEVKNFLESKLPGTQIEVEGEGCNFQLNLISDELAGLSPVK

RQQQIYAHLNEWIASGAIHAVTMKFFSRAAWAERS"

ORIGIN

5382002 catgccgacc acgacgacgc gccccttgca gttcagcaac agttcgcagg ccagcgaaaa

5382062 atcggtgccg atgcgcggca gcagggcgtc caccgagtca cgctcgaggc ggatggtgcg

5382122 ttgtgccgac tggatgaaat cgtgtttctg gctcatgttc gaaaggcgcg gggcaaaatg

5382182 aagtgcgaga ttataacggg aaagcgacaa aggcttcatc actggcaact caatgttgaa

5382242 cgcattttgc tgaactgggc cttaacagcg gcttagcagc ccttggggcg cggcttcacg

5382302 gggtgttata gttcgcggcc gttcggcctg ccgcgccgtg tttctgccgt atgacagacg

5382362 gggcaacgga cagcgccgtg atgaaaggag ttctatgagc accaatgacc aatacgcggt

5382422 cgagctgaag ggcctgagct tcaagcgtgg cacgcgtaag attttcgata acgtggacat

5382482 ccgcattcca cgcggcaagg tcaccgggat catggggccg tcggggtgtg gcaagaccac

5382542 cctgttgcga ctgatcgccg cccaactgcg tccggcgagc ggcgaagtgt gggtcaacgg

5382602 ccagaacctc cccacgctgg ggcgcagcga cctgttcgac atgcgcaagc agttcggcgt

5382662 gttgttccag agcggtgcgc tgttcaccga tctcgatgtc ttcgagaacg ttgccttccc

5382722 gctgcgcgtg cacaccgagc tgcccgagga catgattcgt gacattgtcc tgatgaagct

5382782 ccaggccgtt ggcctgcgcg gggcggtgga actgatgccc gatgagctgt ccggcggtat

5382842 gaagcgccgt gtggcgctgg cccgcgccat cgcgctggac ccgcagatcc tcatgtacga

5382902 cgagcccttc gtggggcagg acccgatcgc catgggcgtg ctggtgcgcc tgatccgcct

5382962 gctcaacgat gccctgggca tcacctccat cgttgtgtcc cacgatctgg cggaaacggc

5383022 gagcatcgcc gactacatct atatcgtcgg tgacggccgt gtgctcggtc acggaacgcc

5383082 ggccgacctg cagggtctcg atgatccgcg agtgcgccag ttcatgaagg gcatcccgga

5383142 tgggccggtg ccgttccact atcctgctcg cgattaccgc gccgacctgc tgggagaacg

5383202 ttgatgcgca agacttcatc gctcgagcga gttcgcctgc tcgggcgcgc gggcctcgat

5383262 gtcctcgagt cgctgggccg ttcgagcctg ttcctggtcc acgccatttt cggccgccgg

5383322 gtgcacagcg gtgccttcca gttgctggtc aaacagctgt attcggtggg tgtgctgtcc

5383382 ctggcaatca tcgtcgtttc cggcctgttc atcggcatgg tgctggcgct gcagggctac

5383442 aacatcctgg cgagctacgg ttcggaacag gcggtcggcc agatggttgc gctgacgctg

5383502 ctgcgtgaac tggggccggt agtcaccggt ctgctgttcg ccgggcgtgc aggctccgcg

5383562 ctgacggccg agatcggcaa catgaaatcc accgagcagt tgtccagcct ggagatgatc

5383622 ggcgtcgacc cgctcaagta catcatcgct ccgcgccttt gggccggctt catctcgatg

5383682 ccgctgctgg cagctatctt cagtgtggtc ggcatctggg gcggcgccct ggtcgccgtg

5383742 gactggctgg gtgtgtatga cggttcgtac tggtcgaaca tgcagaacag cgtgcagttc

5383802 accaaggaca ttctcaatgg cgtgatcaag agtgtggtgt tcgccttcgt cgtgacctgg

5383862 atcgccgtgt tccagggtta tgactgcgaa cccacctcag aggggatcag ccgcgccacc

5383922 acccgtaccg ttgtgtatgc ctccctggcc gtgctggggc tggacttcat cctgaccgcc

5383982 ttgatgtttg gagacttctg atgcaaaccc gcaccctgga aatcggtgta ggcctgttca

5384042 ttctggccgg cctgctggcc ctgctgctgc tggccctgcg tgtcagcgga ctgaccatcg

5384102 gcaattccgg cgatacctac aagctgtatg cctatttcga caacatcgcc ggcgttaccg

5384162 tccgcggaaa ggtcaccatg gcgggtgtca ccatcggcaa ggtcactgcg gtggacctgg

5384222 accatgacag ctatcgtggt cgtgtgacca tggagatcaa tcgtcaggtc aacaacctgc

5384282 cggacgactc taccgcctcc atcctcactg ctggcctgct gggcgagaaa tacatcggca

5384342 tcagtgtcgg tggcgacgaa gagctgctga aggatggcga taccatcaag gacacccagt

5384402 cggcactcgt gctggaagac ctgatcggca aattcctgct gaattcggtc aacaaagacg

5384462 actcgaagaa atgagggctc actccatgtt gaaaaccttg cgccgtggct tcctggtcct

5384522 gctcgcggtc ctgccgctgt tcgcccaggc cgagcagact gctcagcagg tcgtgcagca

5384582 gaccgtcgac agcctgctgt cggacctgaa gaccaacaag gccagctaca aggccaatcc

5384642 ccaggcgttc tacgacaccc tgaaccgcat cctcggcccg gtcgtggatt ccgagggcat

5384702 cgccaagggc gtgatgaccg tcaagtactc ccgccaggcg acgccggagc aggtcaagcg

5384762 cttcgaggaa accttcaaga acagcctgtt ccagttctac ggcaacgcgc tgctggagta

5384822 cgacaaccag gacatccgcg tgctgaacag cggcaagcag gagcaggatc gcgcttcggt

5384882 gaacatggaa gtggtcggca gcaatggcgc cgtctatccg gtgcaataca ccatggtcaa

5384942 ccagggtggc gattggcgcc tgcgtaacgt gatcatcaac ggcatcaaca tcggcaagct

5385002 gttccgtgac cagttcgccg actccatgca gaagaaccgc aacgacctgg agaagaccat

5385062 cgccggttgg ggcctggtcg tcgccaaggc caaggatgcg gccaagggcc agccggaagg

5385122 cagcgcagaa tgagtcaggg cagtatcacg gagcgcagtg agggcgacct ggccctcgcc

5385182 ggcgtgctgg actacaggac cggcccggcg ctgcgcgagc agggcagaag cctgatcgcc

5385242 gccagcaagg ccgcgtcgct gcgcctggac tgctcggcgg tggaacactc cagtagcgtc

5385302 ggattgtcgc tgctgctgtc ctacacccgt gatgcgcgca aggctggcaa gacgctgcag

5385362 atctctggga tgcccaagga catgcaggaa atcgccaagg ttggcgagct tctcgagatt

5385422 ctgccgttac agcattgaaa cagtgagaaa gccctccgtc cgcgacttcc cctgccgggg

5385482 ttcgcaggcg gggggctttt ttgtatcatg gccgacccgc gcgcgtaggg cgccgaacga

5385542 ggttgagcat gcaggccgta gaagtcaaaa acttcctgga atcgaaactg ccaggtaccc

5385602 agatcgaagt ggaaggcgaa ggctgcaatt tccagctgaa cctgatcagc gacgagctcg

5385662 ccggcctgag cccggtcaag cgccagcaac agatctatgc ccacctgaac gagtggatcg

5385722 cctccggcgc catccatgcc gtcaccatga aattcttcag ccgcgccgcc tgggccgagc

5385782 gttcctgagc ccacggggaa attagcaatg gataaactga tcattaccgg cggtactcgt

5385842 ctcgatggcg agattcgcat ctccggcgcg aagaactctg cgctgccgat cctcgccgca

5385902 accctgctgg cggacacccc ggtgaccgtc tgcaacctgc cgcacctgca cgacatcacc

5385962 accatgatcg agctgttcgg ccgcatgggc gtgcagccga tcatcgacga gaagctcaac

5386022 gtcgaagtcg acgccagcac catcaagacc ctggtggcac cgtacgagct ggtgaaaacc

5386082 atgcgtgcgt cgatcctcgt gcttggcccg atggtcgccc gtttcggcga agccgaagtc

5386142 gccctgcccg gcggctgcgc catcggttcg cgtccggttg acctgcacat ccgcggcctt

5386202 gaagccatgg gcgcagaaat cgccgtcgaa ggcggctaca tcaaggccaa ggccccggcc

5386262 ggcggcctgc gcggtgcgca cttcttcttc gataccgtca gcgtgaccgg taccgagaac

5386322 atcatgatgg ccgctgcact ggccaatggc cgctccgtgc tggaaaacgc cgcgcgcgag

5386382 cctgaggtcg tcgacctggc caacttcatc aacgccatgg gcggtgatgt gcagggtgcc

5386442 ggtaccgata ccatcgtgat caacggtgta aaaa //
